# The predictive value of precipitating factors on clinical outcomes in hospitalized patients with decompensated heart failure: insights from the Egyptian cohort in the European Society of Cardiology Heart Failure long-term registry

**DOI:** 10.1186/s43044-023-00342-9

**Published:** 2023-03-08

**Authors:** Ahmed Bendary, Mahmoud Hassanein, Mohamed Bendary, Ahmed Smman, Ahmed Hassanin, Mostafa Elwany

**Affiliations:** 1 Cardiology Department, Faculty of Medicine, Benha University, Benha, Egypt; 2 Cardiology Department, Alexandria University, Alexandria, Egypt; 3 Biostatistics Department, National Cancer Institute, Cairo University, Cairo, Egypt; 4 Alexandria University Students’ Hospital, Alexandria, Egypt; 5 Division of Cardiology, University of Arkansas for Medical Sciences, Arkansas, NY USA

**Keywords:** Heart failure, Precipitating factors, Mortality, Egypt

## Abstract

**Background:**

Knowledge of the frequency of precipitating factors for acute heart failure (AHF) is important (either new-onset heart failure [NOHF] or worsening heart failure [WHF]), as this can guide strategies for prevention and treatment. Most data come only from Western Europe and North America; nevertheless, geographic differences do exist. We set out to study the prevalence of precipitating factors of AHF and their connection to patient characteristics and in-hospital and long-term mortality in patients from Egypt hospitalized for decompensated HF. Using the ESC-HF-LT Registry which is a prospective, multicenter, observational study of patients confessed to cardiology centers in the nations of Europe and the Mediterranean, patients presenting with AHF were recruited from 20 centers all over Egypt. Enrolling physicians were requested to report possible precipitants from among several predefined reasons.

**Results:**

We included 1515 patients (mean age 60 ± 12 years, 69% males). The mean LVEF was 38 ± 11%. Seventy-seven percent of the total population had HFrEF, 9.8% had HFmrEF, and 13.3% had HFpEF. The commonly reported precipitating factors for AHF hospitalization among study population were as follows (in decreasing order of frequency): infection in 30.3% of patients, acute coronary syndrome/myocardial ischemia (ACS/MI) in 26%, anemia in 24.3%, uncontrolled hypertension in 24.2%, atrial fibrillation (AF) in 18.3%, renal dysfunction in 14.6%, and non-compliance in 6.5% of patients. HFpEF patients had significantly higher rates of AF, uncontrolled hypertension, and anemia as precipitants for acute decompensation. ACS/MI were significantly more frequent in patients with HFmrEF. WHF patients had significantly higher rates of infection and non-compliance, whereas new-onset HF patients showed significantly higher rates of ACS/MI and uncontrolled hypertension. One-year follow-up revealed that patients with HFrEF had a significantly higher rate of mortality compared to patients with HFmrEF and HFpEF (28.3%, 19.5, and 19.4%, *P* = 0.004). Patients with WHF had a significantly higher rates of 1-year mortality when compared to those with NOHF (30.0% vs. 20.3%, *P* < 0.001). Renal dysfunction, anemia, and infection were independently connected to worse long-term survival.

**Conclusions:**

Precipitating factors of AHF are frequent and substantially influence outcomes after hospitalization. They should be considered goals for avoiding AHF hospitalization and depicting those at highest risk for short-term mortality.

**Supplementary Information:**

The online version contains supplementary material available at 10.1186/s43044-023-00342-9.

## Background

Heart failure (HF) is one of the major causes of morbidity and mortality [1], and its incidence, prevalence, and economic tolls are increasing. Additionally, HF is the main reason for older patients’ hospitalizations. Hospitalizations for HF may result from a number of established triggering causes [2, 3]. These include arrhythmias, myocardial ischemia, pneumonia, hypertension, deteriorating renal failure, and non-adherence to diet or medication. Importantly, the frequency of precipitating factors varies in different ethnic groups and geographic locations. It is still largely unknown if ascertaining precipitating factors of decompensated heart failure (either new onset or worsening) can depict patients at the highest risk of an unfavorable outcome.

Knowledge on the prevalence of various precipitating factors seems mandatory, as this can drive preventative strategies prior to hospital admission and treatment goals during hospitalization. Moreover, elucidating the relation between precipitating factors and mortality may provide guidance on a category of patients who may need intensification of management strategies during their hospital admission [4–6]. However, there are no studies examining the incidence of these precipitants in Egyptian patients hospitalized with decompensated HF. The study’s purpose was to investigate the prevalence of precipitating factors of AHF and their association with patient attributes and clinical results in the Egyptian patients included in the European Society of Cardiology heart failure long-term (ESC-HF-LT) registry.

## Methods

### Study overview

ESC-HF-LT Registry technique has been described previously [7, 8]. Shortly, this prospective, multicenter observational study involved patients who visited cardiology clinics in numerous European and Mediterranean nations. Twenty locations participated in this registration, including the Mediterranean coast, the Nile Delta, Greater Cairo, Upper Egypt, and the Suez Canal regions. The selection of the centers was made with the intention of having a representative sample of hospitals with varying degrees of complexity. Nine participating locations were university hospitals, and 7 of them lacked cardiac surgery or catheterization laboratories. A total of 1661 Egyptian patients who experienced heart failure and were hospitalized between April 2011 and September 2014 were added to the ESC-HFLT Registry. Patients were followed up by telephone calls at a mean of 1 year after admission for vital status (alive or dead) only. The responsible cardiologist at participating locations made a clinical diagnosis of HF. Patients have to be at least 18 years old to consent to the study. In accordance with the rules established by each participating site, the Registry was authorized by each regional Research Ethics Committee (REC).

The European Society of Cardiology (ESC) department’s EURObservational Research Program (EORP) oversaw the project’s operational management, provided assistance to the participating sites, and kept an eye on the registry’s methodology. Additionally, an auditor chosen by the Steering Committee randomly visited research centers to check for protocol compliance and to review the consecutiveness and quality of data. With the assistance of the EORP department, the database was created at the ESC in accordance with the specifications established by the appointed Steering Committee. The Declaration of Helsinki served as the basis for conducting this study. All patients or, if allowed, a legal representative, provided written informed consent.

### Study patients

All HF patients hospitalized for acute, preexisting, or new-onset HF in participating centers during the enrollment period were considered for the study. Patients were enrolled in the study on a one-day-per-week basis and monitored for at least one year to make consecutive enrollment easier. The single day per week regulation was then altered to five days per season during the registry, as suggested by the study’s executive committee. Acute heart failure (AHF) was outlined as either new-onset heart failure (NOHF) or the decompensation of chronic heart failure (CHF) with symptoms necessitating hospitalization. There were no clear requirements for exclusion other than the age < 18 years old. Between April 2011 and September 2014, data were gathered. Heart failure with reduced ejection fraction (HFrEF ≤ 40%), heart failure with mildly reduced ejection fraction (HFmrEF: 41–49%), and heart failure with preserved ejection fraction (HFpEF ≥ 50%) were further subdivided into the cohort.

### Primary aim

To determine the prevalence of AHF precipitating events and their associations with patient characteristics, in-hospital mortality, and long-term mortality in the Egyptian cohort of patients participating in the ESC-HF long-term Registry.

### Secondary aims(s)

- Identify which precipitating factors are connected to various clinical presentations of HF.
- Explore interactions with subgroups (listed below) for clinical outcome
  ◦ Patients hospitalized for worsening HF vs new-onset HF.
  ◦ Heart failure with reduced ejection fraction (HFrEF) versus heart failure with mildly reduced ejection fraction (HFmrEF) and heart failure with preserved ejection fraction (HFpEF).
  ◦ Hospital length of stay.

### Studied variables

A. *Precipitating factors*: enrolling physicians were asked to report potential precipitating factors from among several predefined reasons: ACS/MI, arrhythmia, infection, uncontrolled hypertension, non-compliance, worsening renal function, and anemia. More than one precipitating factor could be assigned to each patient when applicable, according to the clinician’s judgment.
  The following definitions were applied: ‘ACS/MI,’ as defined by the ESC, in the presence of ECG changes and/or a dynamic rise in standard Troponin readings [9], ‘infection’ in the presence of fever and/or other indications of infection at initial admission (leukocytosis, increased inflammatory markers, clinical or microbiological evidence of infection); ‘atrial fibrillation’ in the presence of AF (new onset or recurrent) with ventricular rate ≥110/min; ‘hypertension’ in the presence of high systolic blood pressure (≥160 mmHg) at admission; ‘anemia’ if hemoglobin level on admission was ≤ 8.0 gm/dl; ‘renal dysfunction’ if serum creatinine level on admission was ≥ 1.5 mg/dl; and ‘non-compliance’ if a significant deviation from nutritional or treatment recommendations is seen (either in patients with a prior diagnosis of HF or in patients who have medical problems that if became uncontrolled due to non-compliance could precipitate HF).
B. In-hospital and long-term all-cause mortality and duration of hospital stay
C. Relevant confounders including demographics (such as age and gender) and signs and symptoms at admission (such as heart failure status, presence or absence of pulmonary edema, and/or cardiogenic shock) and modes of presentation (own transport vs ambulance).

### Statistical analysis

For both data administration and statistical analysis, SPSS version 28 was utilized (IBM, Armonk, New York, USA). Quantitative data were expressed as means and standard deviations, medians, and ranges, while numbers and percentages were used to represent categorical data. One-way ANOVA or the Kruskal–Wallis test was used to evaluate quantitative data according to ejection fraction categories, and independent t tests or the Mann–Whitney U test was used to assess the status of heart failure (new onset versus worsening). Chi-square analysis was utilized to compare all of the categorical data. Post hoc analysis was done for quantitative data when compared between more than two categories, and all post hoc analyses were adjusted for multiple comparisons. Multivariate logistic regression analysis was done for precipitating factors to predict hospital stay of more than 5 days. The odds ratios were estimated with 95% confidence intervals. Overall survival was estimated using Kaplan–Meier analysis and compared according to different precipitating factors using the log-rank test. Multivariate Cox regression analysis was done for different precipitating factors to predict survival. Hazard ratios with 95% confidence intervals were calculated. On the basis of specialist knowledge, multivariable models were chosen. There were two sides to each statistical test. P values under 0.05 were deemed significant.

## Results

### Baseline characteristics

Among the 1661 hospitalization for heart failure (HHF) Egyptian patients, 12 patients were excluded because their unique identifiers were missing, 4 patients from the Suez Canal region who represented the HHF were also disqualified because the group was too tiny to adequately represent the area and 130 patients were excluded because their long-term follow-up data were missing. In total, 1515 patients were left for the final analysis.

The mean age was 60 ± 12 years, with higher male preponderance (69%). The mean LVEF was 38 ± 11%. Seventy-seven percent of the total population had HFrEF with LVEF ≤ 40%, 9.8% had HFmrEF with LVEF ranging from 41 to 49%, and 13.3% had HFpEF with LVEF ≥ 50%. Regarding HF status, 39% had new-onset HF and 61% had worsening HF. Other demographics, cardiovascular risk factors, and comorbidities are illustrated in Table 1.

**Table 1:** General characteristics of the whole population and according to ejection fraction

	All	Ejection fraction (%)			
		< 40	41–49	≥ 50	*P*-value
Age (years)	60 ± 12	61 ± 12 ^a^	62 ± 10 ^a^	58 ± 12 ^b^	< 0.001*
*Gender*					
Males	1046 (69)	858 (73.6)	102 (68.5)	86 (42.8)	< 0.001*
BMI (kg/m^2^)	30.3 ± 5.4	29.6 ± 5.1 ^a^	32 ± 5.2 ^b^	32.6 ± 6.2 ^b^	< 0.001*
HF history	932 (61.5)	758 (65.1)	79 (53)	95 (47.3)	< 0.001*
Smoking	917 (60.5)	755 (64.8)	90 (60.4)	72 (35.8)	0.002*
Hypertension	655 (43.2)	509 (43.7)	61 (40.9)	85 (42.3)	0.782
History of AF	374 (24.7)	257 (22.1)	35 (23.5)	82 (40.8)	< 0.001*
Diabetes	683 (45.1)	541 (46.4)	77 (51.7)	65 (32.3)	< 0.001*
Thyroid disease	27 (1.8)	16 (1.4)	5 (3.4)	6 (3)	0.087
COPD	223 (14.7)	178 (15.3)	15 (10.1)	30 (14.9)	0.239
Stroke	115 (7.6)	86 (7.4)	12 (8.1)	17 (8.5)	0.847
Peripheral vascular disease	79 (5.2)	69 (5.9)	7 (4.7)	3 (1.5)	0.032*
*Heart failure status*					
New onset	586 (38.7)	410 (35.2)	70 (47)	106 (52.7)	< 0.001*
Worsening	929 (61.3)	755 (64.8)	79 (53)	95 (47.3)	
*Primary etiology*					
Ischemic	1028 (67.9)	828 (71.1)	124 (83.2)	76 (37.8)	< 0.001*
Non-ischemic	487 (32.1)	337 (28.9)	25 (16.8)	125 (62.2)	
Baseline Hemoglobin (gm/dl)	11.7 ± 2.1	11.8 ± 2.2	11.7 ± 2.1	11.4 ± 2.2	0.116
Baseline creatinine (md/dl)**	1.2 (0.4–12)	1.2 (0.4–12) ^a^	1.2 (0.5–8) ^a, b^	1.1 (0.5–6.6) ^b^	0.008*

Data were presented as mean ± SD or number (percentage); Different superscripted letters indicate statistically significant differences between both groups; * Asterisk indicates an overall significant difference; ** presented as median (range)

BMI = Body mass index; AF = Atrial fibrillation; HF = Heart failure; and COPD = Chronic obstructive pulmonary disease

Patients with HFrEF and HFmrEF were significantly older than HFpEF patients (*P* < 0.001). Females represented the vast majority of HFpEF patients; however, males represented the vast majority of HFrEF and HFmrEF patients (*P* < 0.001). Mean BMI in patients with HFpEF and HFmrEF was significantly higher than those patients with HFrEF (32.6 ± 6.2, 32 ± 5.2, and 29.6 ± 5.1 kg/m^2^ respectively, *P* < 0.001). Other CV risk factors and comorbidities (including history of AF) differed significantly between various categories of HF (Table 1).

Compared to patients with new-onset HF, those patients with WHF were significantly older (62 ± 12 vs. 59 ± 13 years, P < 0.001) and had significantly lower LVEF (36 ± 11 vs. 41 ± 11, *P* < 0.001), with significantly higher rates of comorbidities (including lower hemoglobin and higher serum creatinine at baseline) (Table 2).

**Table 2:** General characteristics according to heart failure status

	HF status		*P*-value
	New onset (586)	Worsening (929)	
Age (years)	59 ± 13	62 ± 12	< 0.001*
*Gender*			
Males	389 (66.4)	657 (70.7)	0.075
Females	197 (33.6)	272 (29.3)	
BMI (kg/m^2^)	31.1 ± 5.5	29.7 ± 5.2	< 0.001*
Smoking	323 (55.1)	594 (63.9)	< 0.001*
Hypertension	253 (43.2)	402 (43.3)	0.970
History of AF	112 (19.1)	262 (28.2)	< 0.001*
Diabetes	234 (39.9)	449 (48.3)	0.001*
Thyroid disease	11 (1.9)	16 (1.7)	0.824
COPD	59 (10.1)	164 (17.7)	< 0.001*
Stroke	36 (6.1)	79 (8.5)	0.091
Peripheral vascular disease	12 (2)	67 (7.2)	< 0.001*
*Primary etiology*			
Ischemic	408 (69.6)	620 (66.7)	0.241
Non-ischemic	178 (30.4)	309 (33.3)	
Ejection fraction (%)	41 ± 11	36 ± 11	< 0.001*
Baseline Hemoglobin (gm/dl)	11.9 ± 2.2	11.6 ± 2.1	0.002*
Baseline creatinine (mg/dl) **	1.1 (0.4–12)	1.2 (0.5–8.2)	< 0.001*

*BMI* Body mass index; *AF* Atrial fibrillation; *HF* Heart failure; and *COPD* Chronic obstructive pulmonary disease

*Significant; Data were presented as mean ± SD or number (percentage); and ** presented as median (range)

### Precipitating factors for HF

The most reported precipitating factors for HF hospitalization among study population were as follows (in decreasing order of frequency): infection in 30.3% of patients, ACS/MI in 26%, anemia in 24.3%, uncontrolled hypertension in 24.2%, AF in 18.3%, renal dysfunction in 14.6%, and non-compliance in 6.5% of patients. About 35% of the study population had only one precipitating factor, 27% had two precipitating factors, and 16% had more than two precipitating factors, and 22% did not have any apparent precipitating factor.

ACS/MI, AF, anemia, and uncontrolled hypertension differed significantly between different types of heart failure (HFrEF, HFmrEF, and HFpEF). On the other hand, all types of heart failure showed similar rates of infection, renal dysfunction, and non-compliance as precipitating factors (Table 3 and Additional file 1: Fig. S1).

**Table 3:** Precipitating factors for the whole population and according to ejection fraction and HF status (new onset versus worsening)

	All	HF status			Ejection fraction (%)			
		New onset	Worsening	*P*-value	< 40	41–49	≥ 50	*P*-value
ACS/MI	394 (26)	261 (44.5)	133 (14.3)	< 0.001*	282 (24.2)	68 (45.6)	44 (21.9)	< 0.001*
Atrial fibrillation	277 (18.3)	100 (17.1)	177 (19.1)	0.330	191 (16.4)	25 (16.8)	61 (30.3)	< 0.001*
Infection	459 (30.3)	137 (23.4)	322 (34.7)	< 0.001*	365 (31.3)	33 (22.1)	61 (30.3)	0.072
Non-compliance	98 (6.5)	23 (3.9)	75 (8.1)	0.001*	80 (6.9)	8 (5.4)	10 (5)	0.511
Uncontrolled hypertension	367 (24.2)	199 (34)	168 (18.1)	< 0.001*	215 (18.5)	57 (38.3)	95 (47.3)	< 0.001*
Renal dysfunction	221 (14.6)	77 (13.1)	144 (15.5)	0.205	164 (14.1)	26 (17.4)	31 (15.4)	0.513
Anemia	368 (24.3)	128 (21.8)	240 (25.8)	0.078	258 (22.1)	43 (28.9)	67 (33.3)	0.001*

^*^ Significant; Data were presented as number (percentage)

ACS/MI = Acute coronary syndrome/Myocardial ischemia

Compared to new-onset HF, WHF patients showed a significantly higher rates of infection (34.7% vs 23.4%, *P* < 0.001) and non-compliance (8.1% vs. 3.9%, *P* = 0.001). On the other hand, new-onset HF patients showed a significantly higher rates of ACS/MI (44.5 vs 14.3, *P* < 0.001) and uncontrolled hypertension (34% vs. 18.1, *P* < 0.001) as precipitating factors. Other precipitating factors did not differ significantly between new-onset and WHF patients (Table 3).

### Predictive value of precipitating factors for in-hospital outcome

With a range of 0–98 days, the median hospital stay was 5 days. About half of the patients (718 patients, 47.4%) had hospital stay of more than the median (5 days). Patients with HFrEF showed similar rates of in-hospital mortality compared to those patients with HFmrEF and HFpEF (5.1%, 4.7%, and 5.0% respectively, *P* = 0.981). There was also no statistically significant difference between patients with new-onset and patients with WHF in terms of in-hospital mortality (5.3% vs.. 4.8%, *P* = 0.698). A multivariate logistic regression analysis (adjusted for age, gender, HF history, AF history, and HF status) showed that non-compliance as a precipitating factor was an independent predictor for a prolonged hospital stay more than the median (5 days), OR (95% CI) [1.576 (1.032–2.406)], *P*-value = 0.035. On the other hand, the following precipitating factors were associated with a significantly shorter hospital stay less than the median: ACS/MI, OR (95% CI) [0.545 (0.421–0.706)], *P*-value < 0.001, atrial fibrillation, OR (95% CI) [0.572 (0.374–0.876)], *P*-value = 0.01, and uncontrolled hypertension OR (95% CI) [0.328 (0.251–0.429)], *P*-value < 0.001.

In-hospital mortality was reported in 76 patients (representing 5% of the whole study population). A multivariate logistic regression analysis (adjusted for age, gender, HF history, AF history, and HF status) showed that the following precipitating factors were independent predictors for in-hospital mortality: anemia, HR (95% CI) [1.736 (1.062–2.837)], *P*-value = 0.028, and renal dysfunction, HR (95% CI) [2.746 (1.534–4.616)], *P*-value < 0.001 (Table 4).

**Table 4:** Multivariate cox regression analysis for precipitating factors to predict overall survival of the studied patients

	HR for in-hospital mortality (95% CI)**	*P*-value	HR for long-term mortality (95% CI)**	*P*-value
ACS/MI	0.983 (0.55–1.758)	0.955	0.703 (0.542–0.911)	0.008*
Atrial fibrillation	0.489 (0.183–1.304)	0.153	0.613 (0.409–0.92)	0.018*
Infection	1.37 (0.853–2.199)	0.193	1.317 (1.07–1.622)	0.009*
Non-compliance	0.66 (0.207–2.107)	0.483	0.393 (0.221–0.7)	0.002*
Uncontrolled hypertension	0.247 (0.094–0.646)	0.004*	0.702 (0.548–0.9)	0.005*
Renal dysfunction	2.746 (1.634–4.616)	< 0.001*	1.875 (1.481–2.374)	< 0.001*
Anemia	1.736 (1.062–2.837)	0.028*	1.765 (1.425–2.185)	< 0.001*

*HR* Hazard ratio; *95% CI* 95% confidence interval

*Significant; **Adjusted for age, gender, HF history, AF history, and HF status

### Predictive value of precipitating factors for long-term mortality

**Fig. 1:**
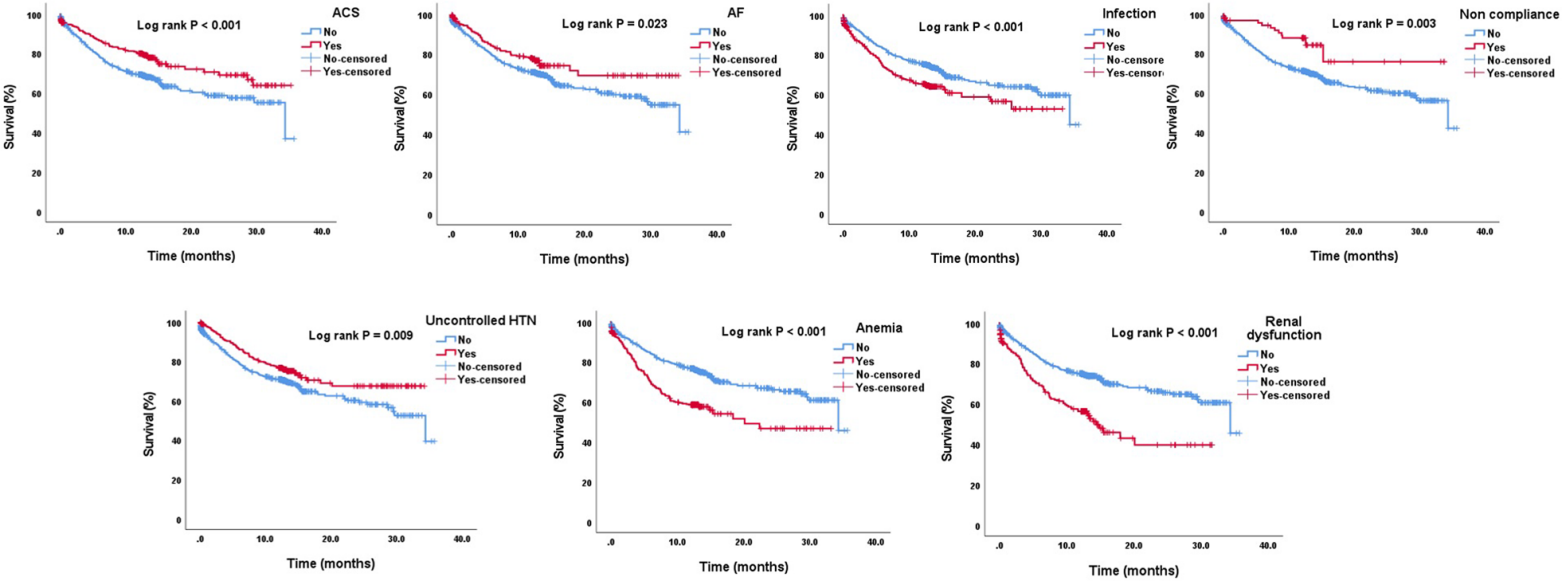
Overall survival according to different precipitating factors. ACS = Acute coronary syndrome; AF = Atrial fibrillation; HTN = Hypertension

**Fig. 2:**
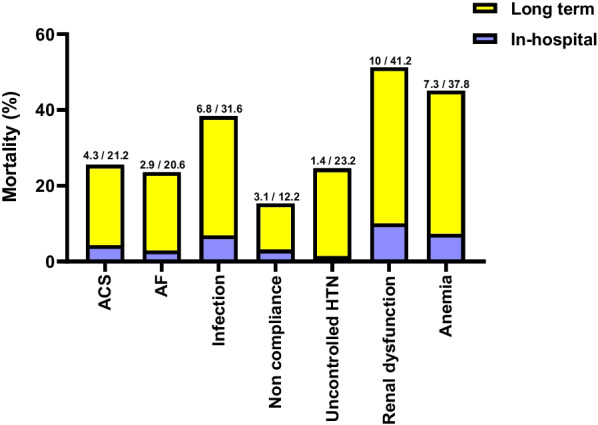
In-hospital and long-term mortality rates for each precipitating factor are represented above each column. ACS = Acute coronary syndrome; AF = Atrial fibrillation; and HTN = Hypertension

The median overall survival time was 34.4 months, with 95% CI ranging from 26.2–42.5 months. Mortality rates during follow-up were reported to be 27.8% at 12 months, 35% at 18 months, 38% at 24 months, and 42.8% at 30 months. A landmark analysis at 1 year follow-up showed that patients with HFrEF had a significantly higher rate of mortality compared to patients with HFmrEF and HFpEF (28.3%, 19.5, and 19.4%, *P* = 0.004). Patients with worsening HF had a significantly higher rates of 1-year mortality when compared to those with new-onset HF (30.0% vs 20.3%, *P* < 0.001). A multivariate cox regression analysis (adjusted for age, gender, HF history, AF history, and HF status) for prediction of survival showed that renal dysfunction, anemia, and infection were associated with worse long-term survival. On the other hand, uncontrolled hypertension, AF, non-compliance, and ACS/MI were associated with better long-term survival (Table 4 and Fig. 1). Rates for both in-hospital and long-term mortality for *each* precipitating factor are illustrated in Fig. 2.

## Discussion

Prior studies on the prevalence and connection to mortality of precipitating factors for patients admitted for AHF were largely from North America and Western Europe [10–12]. This is the first in time study, as far as we know, to examine the precipitants for acute decompensation of heart failure in a large cohort of Egyptian patients hospitalized for AHF and their association with mortality.

The most reported precipitating factors for HF hospitalization among our study population were—in decreasing order of frequency—infection, acute coronary syndrome, anemia, uncontrolled hypertension, atrial fibrillation, renal dysfunction, and non-compliance. In the Get with The Guidelines-HF (GWTG-HF) database [10], two or more factors were identified in 26.7% and 3 or more factors in 8.1%; pneumonia/respiratory process (28.2%), arrhythmia (21.7%), medication non-compliance (15.8%), deteriorating renal failure (14.7%), and uncontrolled hypertension (14.5%) were shown to be the most prevalent precipitants potentially contributing to AHF. Berkovitch A et al. [13] examined 2212 individuals who had been admitted with a diagnosis of either acute decompensation of preexisting HF or new-onset HF. Myocardial ischemia as a precipitating factor was identified in 46% of patients. Major precipitants other than myocardial ischemia were infection (21%), non-compliance (17%), renal dysfunction (13%), and other minor factors (49%). In the GREAT registry [5], ACS was the most frequent cause among the 7764 patients who had just one precipitant identified (52%), followed by AF (16%), infection (14%), uncontrolled hypertension (11%), and non-compliance (8%). In the CHARM Program [12], among the non-cardiovascular causes for hospitalization, respiratory tract infection was the commonest single factor. It can be perceived that precipitants for AHF look similar among different populations; however, their hierarchy differs. This is attributed to geographic variation of the population studied, multiplicity of risk factors in the same individual, and clinical discretion of the attending physician who had to assign the patient to the most plausible precipitating factor. The multiplicity of precipitating risk factors for AHF and their common association may underly the complexity of their relation to early and late mortality.

All HF phenotypes in our study showed similar rates of infection, renal dysfunction, and non-compliance as precipitating factors. However, atrial fibrillation, uncontrolled hypertension, and anemia were considerably more common in patients with HFpEF and served as precipitants for acute decompensation. ACS/MI were much more prevalent among patients with HFmrEF. In the REPORT-HF registry [14], patients with HFmrEF from the Eastern Mediterranean or African region had the highest incidence of ACS/MI. In the same registry, those with HFpEF had more arrhythmias, uncontrolled hypertension, respiratory tract infections, and pulmonary embolism as possible precipitating factors than patients with HFrEF. In the GWTG-HF study [10], patients with greater ejection fraction had higher propensity to have poor control of the blood pressure, less medication adherence, and ACS compared to those who had a lower ejection fraction. Relative to the other groups in the same study, dietary and drug non-compliance were identified with higher frequency in patients with lower EF.

In our study, WHF patients had a significantly higher rates of infection and non-compliance as precipitants of HF. On the other hand, new-onset HF patients showed significantly higher rates of ACS and uncontrolled hypertension. Akin to our results, patients with new-onset HF in the whole cohort of REPORT-HF registry [14] most frequently presented with ACS/MI (18%), arrhythmias (11%), and uncontrolled hypertension (8%), whereas patients hospitalized for non-compliance to diet/medication or medication that can aggravate HF had a significantly higher chances for having WHF. In the Gulf CARE (Gulf aCute heArt failuRe rEgistry) [15], the most frequent precipitating factor in the new-onset HF category was acute myocardial ischemia (39.2%). In the chronic HF group, it was non-adherence with the drugs (27.8%).

We showed that non-compliance is an independent predictor of prolonged hospital stay, in contrast to uncontrolled hypertension and ACS which were associated with shorter stays. Even though this could appear confusing in the 1^st^ sight, we can explain this by the fact that patients admitted with a precipitating factor of non-compliance were significantly more likely to be admitted for worsening HF than new-onset HF. In contrast, patients admitted with a precipitating factor of uncontrolled hypertension or ACS were significantly more likely to be admitted with new-onset HF than worsening HF (Table 3). It is known that patients admitted with worsening HF require more augmented and advanced intensive medical care than those patients admitted for new-onset HF [12], which may have translated into a more prolonged hospital stay.

Our data show that renal dysfunction and anemia were separate predictors of in-hospital mortality, whereas long-term mortality was independently associated with renal dysfunction, anemia, and infection. According to the REPORT-HF registry [14], hospitalized patients with declining renal function had the highest rates of mortality both during their stay and one year after release. In patients with HF decompensations, renal impairment is a well-known independent prognostic factor for negative long-term effects [16]. The factors that may be contributing to the decreasing renal function in HF patients include reduced cardiac output, renal artery hypoperfusion, venous congestion, and increased intraabdominal pressure.

Renal dysfunction and infection, as precipitating factors, were found by Berkovitch et al. [13] to be independently related to higher risk for both early and late mortality. This finding supports the research conducted by Alon et al. [17] who demonstrated higher death rates for HF patients after 30 days and a year after admission for infectious causes. The reason why hospital deaths are more common is obvious, but it is still unclear why a single incident has an impact on long-term survival. In addition, Yende S et al. [18] found admission due to pneumonia to be an independent predictor for long-term cardiovascular events and attribute this higher risk to pro-inflammatory derangements and greater comorbidity burden.

Prevalence of anemia was very high in Egyptian patients admitted with heart failure. El-Sahn et al. [19] demonstrated a general prevalence of anemia of 46.6% among adolescents in Egypt. Another research work revealed prevalence of anemia of 49.6% among attendants of family planning centers in Egypt [20]. A systematic review and meta-analysis by Groenveld HF et al. [21] to evaluate the effect of anemia on mortality in chronic heart failure revealed a rough mortality risk of anemia of odds ratio 1.96 (95% confidence interval: 1.74–2.21, *P* < 0.001). At baseline, lower hemoglobin concentrations were linked to higher crude death rates (r = −0.396, *P* = 0.025).

One-year follow-up in our registry demonstrated those with HFrEF had a significantly higher rate of mortality compared to patients with HFmrEF and HFpEF. This is consistent with a patient-level meta-analysis showing a reduced risk of death in HFpEF compared to HFrEF [22]. When compared to patients with new-onset HF, those in the Egyptian registry with WHF had significantly higher 1-year mortality rates. Using information from national Danish registries, Butt JH et al. [23] discovered that when compared to HF that developed suddenly, preexisting HF had a greater rate of the composite outcome of all-cause death or HF readmission.

Uncontrolled hypertension, AF, non-compliance, and ACS were connected to reduced long-term mortality in our patients. According to our analysis, there was a comparatively low risk of death when AF caused AHF. These results are in concordance with those of the GREAT registry [5] and the Spanish PAPRICA-2 [24] study, which demonstrated a somewhat positive short-term outcome in AF-induced AHF. In OPTIMIZE-HF [2], the lowest death rate was linked to uncontrolled blood pressure, while those with ACS/MI or declining renal function had the highest mortality rates. On the other hand, the data of Berkovitch et al. [13] indicated that the possibility of 10-year mortality rate was significantly greater among patients with a non-ischemic compared to those with ischemic precipitating factors. The Gulf CARE investigators [5] found that ACS was linked to higher in-hospital and 1-year mortality, but medication non-compliance was linked to decreased in-hospital mortality. The discrepancy between other studies [2, 5, 10, 15] showing increased mortality when ACS was the precipitant of AHF and our registry may be attributed to multiple factors. First, our patients were much younger than patients recruited from Western populations. Second, comorbidities were less prevalent in HF patients from Egypt. Third, coronary angiography was scarcely performed during index hospitalization in our patients; thus, there was no proof of the severity of coronary artery disease.

The current report has some limitations; medical chart reviews were used to collect the data, which depended on accurate and thorough documentation and abstraction. In addition, the assessment of precipitants for decompensated HF was left to the discretion of the enrolling investigators. Precipitants were not mutually exclusive. The local provider determined the major precipitant. Patients could be hospitalized due to more than one precipitating factor. Actually, many patients had more than one precipitant of decompensation. As in any observational study, some unmeasured confounders might have interfered with the results. Furthermore, non-adherence with medication and/or dietary advice might be more common than is often believed, and it might have been greatly influenced by cultural diversity. We are also aware of the limitation of not including data on rehospitalization in the current report. These data were not readily available specifically for the Egyptian cohort of the ESC long-term HF registry at time of manuscript drafting. We are planning to publish this data in the near future. The design of the current study is more like a registry than a prospective longitudinal cohort study. Accordingly (and apart from rehospitalization data), other accumulating/cumulative events occurring during the 1-year period till the follow-up were not recorded and therefore were not accounted for, and this might have influenced the results. Finally, despite the fact that this is a registry-based study which allows to investigate patients in real-world environment, data gathering was dependent on voluntary participation of centers such that it may affect the external validity and generalizability to other centers with different care patterns or patient criteria.

As a future perspective, knowledge of different precipitating factors for hospitalization for AHF highlights potentially important goals for preventing AHF hospital admission and improvement of survival. Controlling hypertension has a great influence on minimizing the risk of incident HF and HF hospitalization, especially in the elderly. Ischemic heart disease was the principal primary etiology of HF in the Egyptian cohort of patients enrolled in the ESC HF long-term registry. Thus, control of risk factors for coronary heart disease would certainly reduce the incidence of HF. The practice of seasonal influenza vaccination may mitigate the deleterious effects of infection and reduce mortality in patients hospitalized for HF. Policies to improve drug compliance among HF patients have significant effects on decreasing rates of rehospitalization and decreasing mortality. Drug adherence should be evaluated in routine follow‐up visits with HF patients, and interventions to improve compliance should be a key part of any quality improving initiative.

## Conclusions

The common precipitating factors for HF hospitalization in Egyptian patients were infection, acute coronary syndrome, anemia, uncontrolled hypertension, atrial fibrillation, renal dysfunction, and non-compliance. WHF patients had higher rates of infection and non-compliance as precipitants of HF, whereas new-onset HF patients showed higher rates of ACS and uncontrolled hypertension.

## Supplementary Information

**Additional file 1**: **Fig. S1** Precipitating factors according to ejection fraction (A) and HF status (B). ACS= Acute coronary syndrome; LVEF= Left ventricular ejection fraction.

## Data Availability

All data are available upon request from the corresponding author(s).

## Abbreviations

ACS/MI: Acute coronary syndrome/myocardial infarction
AHF: Acute heart failure
EORP: EURObservational Research Programme
ESC: European society of cardiology
GWTG-HF: Get with the guidelines heart failure study
HFmrEF: Heart failure with mildly reduced ejection fraction
HFpEF: Heart failure with preserved ejection fraction
HFrEF: Heart failure with reduced ejection fraction
NOHF: New-onset heart failure
OPTIMIZE-HF: Organized program to initiate lifesaving treatment in hospitalized patients with heart failure
WHF: Worsening heart failure
