# Correction: The predictive value of precipitating factors on clinical outcomes in hospitalized patients with decompensated heart failure: insights from the Egyptian cohort in the European Society of Cardiology Heart Failure long-term registry

**DOI:** 10.1186/s43044-023-00346-5

**Published:** 2023-03-13

**Authors:** Ahmed Bendary, Mahmoud Hassanein, Mohamed Bendary, Ahmed Smman, Ahmed Hassanin, Mostafa Elwany

**Affiliations:** 1 Cardiology Department, Faculty of Medicine, Benha University, Benha, Egypt; 2 Cardiology Department, Alexandria University, Alexandria, Egypt; 3 Biostatistics Department, National Cancer Institute, Cairo University, Cairo, Egypt; 4 Alexandria University Students’ Hospital, Alexandria, Egypt; 5 Division of Cardiology, University of Arkansas for Medical Sciences, Arkansas, NY USA

## Correction: The Egyptian Heart Journal (2023) 75:16 https://doi.org/10.1186/s43044-023-00342-9

Following publication of the original article [1], the authors noticed an error in affiliation 3. The correct affiliation is:

Biostatistics Department, National Cancer Institute, Cairo University, Cairo, Egypt.

The original article [1] has been corrected.
