# Supplementary material for: The predictive value of precipitating factors on clinical outcomes in hospitalized patients with decompensated heart failure: insights from the Egyptian cohort in the European Society of Cardiology Heart Failure long-term registry
Source: Egypt Heart J. 2023 Mar 8;75:16. doi: 10.1186/s43044-023-00342-9 (PMC9995627; doi:10.1186/s43044-023-00342-9)
Supplement: Supplementary file 1 — Additional file 1: Fig. S1 Precipitating factors according to ejection fraction (A) and HF status (B). ACS= Acute coronary syndrome; LVEF= Left ventricular ejection fraction. [file 43044_2023_342_MOESM1_ESM.docx]

**Supplementary materials**

Supplementary Figure 1

Precipitating factors according to ejection fraction (A) and HF status (B)


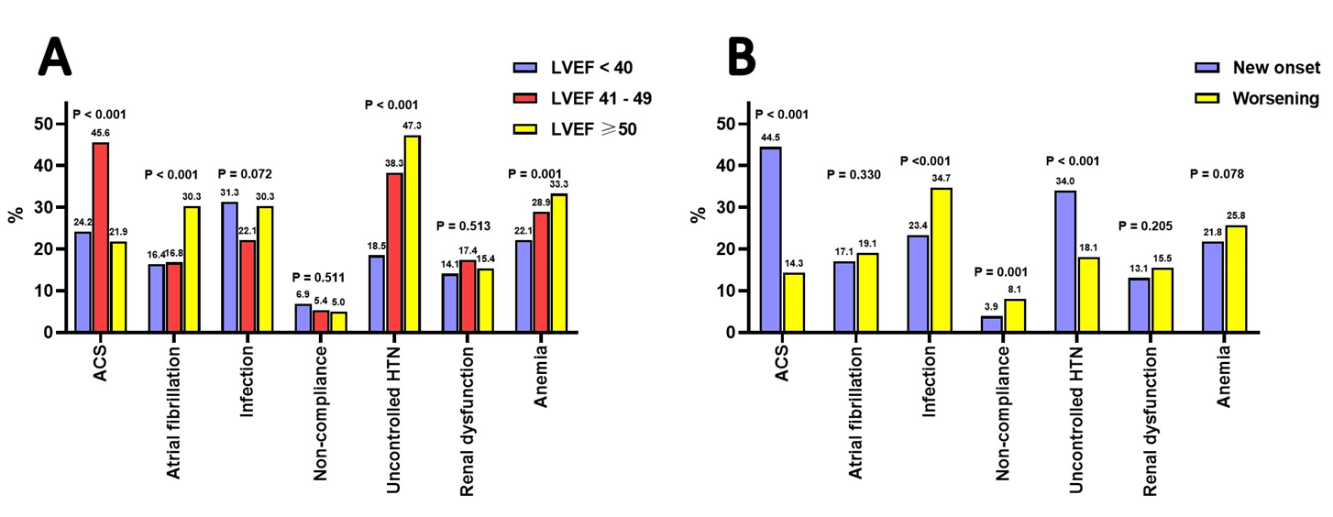


ACS= Acute coronary syndrome; LVEF= Left ventricular ejection fraction
